# Probability-Based LIDAR–Camera Calibration Considering Target Positions and Parameter Evaluation Using a Data Fusion Map

**DOI:** 10.3390/s24123981

**Published:** 2024-06-19

**Authors:** Ryuhei Yamada, Yuichi Yaguchi

**Affiliations:** Department of Computer Science and Engineering, The University of Aizu, Tsuruga, Ikki-Machi, Aizu-Wakamatsu 965-8580, Fukushima, Japan; yaguchi@u-aizu.ac.jp

**Keywords:** extrinsic calibration, LIDAR, camera, calibration target, data fusion

## Abstract

The data fusion of a 3-D light detection and ranging (LIDAR) point cloud and a camera image during the creation of a 3-D map is important because it enables more efficient object classification by autonomous mobile robots and facilitates the construction of a fine 3-D model. The principle behind data fusion is the accurate estimation of the LIDAR–camera’s external parameters through extrinsic calibration. Although several studies have proposed the use of multiple calibration targets or poses for precise extrinsic calibration, no study has clearly defined the relationship between the target positions and the data fusion accuracy. Here, we strictly investigated the effects of the deployment of calibration targets on data fusion and proposed the key factors to consider in the deployment of the targets in extrinsic calibration. Thereafter, we applied a probability method to perform a global and robust sampling of the camera external parameters. Subsequently, we proposed an evaluation method for the parameters, which utilizes the color ratio of the 3-D colored point cloud map. The derived probability density confirmed the good performance of the deployment method in estimating the camera external parameters. Additionally, the evaluation quantitatively confirmed the effectiveness of our deployments of the calibration targets in achieving high-accuracy data fusion compared with the results obtained using the previous methods.

## 1. Introduction

Three-dimensional (3-D) mapping is essential for a mobile robot to perform several tasks, including surrounding environment identification, self-location estimation, object recognition, and autonomous navigation. The simultaneous localization and mapping (SLAM) algorithm is the principal method for constructing a 3-D map of the surroundings of a mobile robot using onboard sensor data, such as camera images and 3-D light detection and ranging (LIDAR) point clouds. The LIDAR SLAM algorithm can provide a wide and accurate 3-D map [1,2,3]. However, a 3-D map constructed from the LIDAR point cloud conventionally has a low resolution and no color information. The map obtained using only point clouds may be insufficient for the detailed identification of environments and objects around a robot. As a solution, data fusion of the 3-D LIDAR point clouds and the camera images is generally implemented to construct a map with detailed information. A camera image can provide color and texture data, enhancing a LIDAR point cloud map. Notably, the data fusion method is highly effective for the classification and identification of multiple objects around autonomous vehicles [4,5,6,7], as well as the construction of fine subcentimeter 3-D models [8].

The requirement for achieving effective data fusion is accurate data matching of the camera image and the LIDAR point cloud. Data matching requires knowledge of the appropriate camera external parameters, which represent the transformation between the coordinate frames of the LIDAR and the camera and the camera intrinsic parameters corresponding to the camera specifications. Moreover, LIDAR–camera calibration (extrinsic calibration) is required to estimate the camera external parameters, for which many methods have been proposed. These extrinsic calibration methods can be classified into two groups: target and targetless methods. Here, we concentrate on the target methods. In target methods, one or more calibration targets are employed, which are observed by the LIDAR and camera simultaneously. The feature points of the 2-D image and the 3-D point clouds of the targets are extracted, and the 3-D feature points are projected on the 2-D image using the camera intrinsic and external parameters. In the extrinsic calibration, the camera external parameters are estimated by matching the 3-D feature points projected on the 2-D plane to those in the 2-D image. The key to achieving accurate extrinsic calibration is the utilization of multiple targets or multiple poses of a single target [9,10]. However, no study has established a clear relationship between the positions of the multiple targets and/or poses and those of the objects used in data fusion quantitatively. Clarifying the effects of the calibration target deployments on data fusion results will provide insights for deploying calibration targets during extrinsic calibration.

Another key factor for achieving precise extrinsic calibration is the estimation method of the camera external parameters. Conventionally, the external parameters are estimated using the least squares method to reduce the differences between the feature points on the 2-D image and the same points projected from the 3-D LIDAR data. This is a nonlinear inversion problem for parameter optimization. Most studies related to extrinsic calibration [11,12] utilize the Levenberg–Marquardt [13] or Gauss–Newton [14] method to solve the nonlinear optimization problem. However, these least-square-based methods tend to derive the local minimum, and insufficient parameter sampling may prevent the determination of the best parameter.

Thus, the estimated camera external parameters should be practically applied to the LIDAR–camera data fusion in the real 3-D world, e.g., outdoor environments, and the parameters should be evaluated in environments similar to a real scenario quantitatively. However, most evaluations of the camera external parameters are performed in simulators and/or limited laboratory spaces [15,16,17]. While some data fusion experiments are performed in real roads and/or broad outdoor areas, they are mostly qualitative [12,18,19].

Therefore, in this paper, we propose three concepts regarding extrinsic calibration.

First, we reveal the relationship between the positions of the calibration targets in the extrinsic calibration and the data fusion accuracy, considering the positions quantitatively. This will enable the optimization of the data fusion of the objects set at specified positions and provide useful information regarding the deployment of the calibration targets during calibration.

Second, we propose a probability-based method for estimating the camera external parameters, i.e., the Monte-Carlo Markov-chain method. This method enables robust parameter sampling and the selection of appropriate parameters from a global search. Thus, our proposed method can be useful when working with a sparse 3-D LIDAR point cloud.

Third, we propose a method for evaluating the adaptability of the camera external parameters obtained by the extrinsic calibration. This method uses a 3-D colored data fusion map obtained in a real outdoor environment. The evaluation result is useful for assessing the practicality of the camera external parameters in more realistic situations.

The remainder of this paper is organized as follows. In Section 2, we discuss related works on extrinsic calibration and expound on the novelty of our concepts. Thereafter, the proposed probability-based methods for the extrinsic calibration and estimation of the camera external parameters using the probability function are explained in Section 3. In Section 4, we describe our measurement system and the experimental conditions under which the appropriate deployment approach for the calibration targets is verified. The camera external parameters estimated using our probability-based method are presented in Section 5. In Section 6, we discuss the relationship between the positions of the calibration targets and the accuracies of the data fusion of objects, considering the positions in the 3-D colored data fusion map. Next, the quantitative method in a 3-D space is applied to evaluate the data fusion results, and the deployment methods for the calibration targets are verified. Finally, the proposed concepts are discussed in Section 8, and the paper is concluded in Section 9.

## 2. Related Works

In this section, we introduce several existing extrinsic calibration methods and expound on the novelty of our proposed concepts. In target extrinsic calibration methods, several types of artificial targets can be employed. The checkerboard is the most used target [11,12,15,16,17,18,20,21,22,23,24]. Zhang and Pless [11] proposed a classical method using a single checkerboard to estimate the camera external parameters. The several poses of the checkerboard observed by a 2-D laser range finder and a camera provided geometric constraints on the rigid transformation between the camera coordinate system and the laser coordinate system. Although some calibration methods using 2-D LIDAR, similar to that of Zhang and Pless [11], have been proposed [20,21], Unnikrishnan and Herbert [18] were the first to apply a 3-D laser scanner for extrinsic calibration using a checkerboard. Therefore, most studies employed 3-D LIDAR. A single checkerboard with several poses [15,16,17] or additional objects, such as hollows [22] and reflectors [12], have been employed for extrinsic calibration, and the method based on Zhoug et al. [23] made available recently in the Matlab R2021a open-source software [25]. Conversely, Ginger et al. [9] and Fu et al. [24] proposed calibration methods using multiple checkerboards. Although the multiple target method requires more time compared to the single board one for the setting, it can provide strict constraints on the LIDAR–camera transformation using only single-view data [9].

Apart from the checkerboard, several calibration targets have also been applied, such as planar square boards [19,26,27], triangle boards [28], spheres [29], 3-D boxes [30,31], and a square board with reflected markers [32]. The combination of planar boards and AR markers [33] or checkerboards [34] has also been researched. A planar board with circles is often applied as a calibration target next to a checkerboard [10,35,36,37,38,39]. Velas et al. [37] confirmed that circular halls on planer markers can be distinguished, even if horizontally oriented edges of other geometrical shapes, such as squares and triangles, cannot be distinguished in 3-D LIDAR point clouds using a single point of view. Thus, circles can also be restored even if only the center and a part of a circle are detected in sparse point clouds. Beltrain et al. [10] and Yamada and Yaguchi [39] demonstrated the usefulness of multiple poses and/or multiple targets using a planar board with circular holes. While many studies related to target methods have indicated that the utilization of multiple poses and/or multiple targets is useful for obtaining optimized camera external parameters, no study strictly focuses on the position of each calibration target. For this reason, using multiple planar boards with circular holes, we investigated the relationship between the positions of the calibration targets and the accuracy of the data fusion considering the object’s positions.

Target-less methods do not require any special artificial targets. Scramuzza et al. [40] used a natural scene, and the features of the scene highlighted on both the camera images and the 3-D range information for the calibration were consistent. The reflectance information measured by the 3-D laser and the grayscale image are also utilized. The transformations between the LIDAR and the camera are examined to maximize the mutual information between the sensor-measured surface intensities [41,42,43,44,45]. Some researchers [46,47,48] have proposed a method based on line information. The line features extracted from objects in office floors [46], buildings [47], and road scenes [48] from both the camera and the 3-D LIDAR data are utilized to derive the camera external parameters. Recent targetless methods also use the semantic information extracted from both 3-D point clouds and camera images [49,50]. Although these targetless methods are not time-consuming and can achieve high calibration accuracy comparable to that of the target methods, they can be affected by the surrounding environments and data acquisition conditions (light and weather conditions). In addition, recent advances in deep learning have provided new methods for LIDAR–camera calibration, including RegNet [51], CalibNet [52], and LCCNet [53]. However, these methods conventionally require a large amount of accurately labeled data pairs for training [10,12], and they consume more time than the simple methods. For these reasons, we focus on target methods.

Although many studies [11,23] utilize traditional methods, such as the Levenberg–Marquart method, in the analysis of the calibration data, we adopt the Monte-Carlo Markov-chain algorithm [54] to solve the nonlinear inverse problem. Our selected algorithm performs a global search of the model parameters by sampling the parameters using the random walk method based on probability. It provides the result of the parameter search as a posteriori probability density and prevents the derivation of a local minimum. Since the algorithms are already implemented to solve complicated nonlinear inversion problems in some geophysical studies [55,56,57], we first adopt the Monte-Carlo Markov-chain algorithm for a more robust search of the camera external parameters.

Using the obtained camera external parameters as the result of the extrinsic calibration, 3-D point clouds with RGB values can be generated, and some related studies have proposed examples of 3-D colored point cloud maps [18,40,41,43]. However, these fusion maps are created in the laboratory and the limited outdoor areas and none of these studies utilized the color information in the fusion data to evaluate the goodness of the derived camera external parameters. The 3-D colored point cloud map is a product of data fusion, and the completeness of the fusion map should be an important factor in validating the goodness of the estimated camera external parameters. We propose an evaluation method that involves using the color information in the 3-D colored point cloud in outdoor areas similar to realistic scenarios.

## 3. Methods

Here, we provide more information on our extrinsic calibration approach, encompassing the method for estimating the camera external parameters, the calibration target adopted, and the method for extracting the feature points from the target. Thereafter, the design of the likelihood function and the parameter sampling using the Monte-Carlo Markov-chain algorithm are explained.

### 3.1. Estimation of the Camera External Parameters

Following Zhang and Press [11] and Cai et al. [22], a pixel coordinate of a point, $p={\left[u,v,1\right]}^{T}$, in a camera coordinate system and a coordinate of a point, $P={\left[x,y,z\right]}^{T}$, in a LIDAR coordinate system can be represented as follows:

(1) sp=K(RP+t),

where K is the camera intrinsic parameter; R is a 3 × 3 rotation matrix, which represents the angular rotation relationship between the two coordinate systems; t is a 3 × 1 translation vector, which represents the relative positional relationship between the two coordinate system; and s is the projective transformation’s arbitrary scaling factor, which is not part of the camera model. Here, the camera intrinsic parameter is represented as follows:

(2) $$K=\left[\begin{array}{ccc} f_{u} & 0 & O_{u}\\ 0 & f_{v} & O_{v}\\ 0 & 0 & 1 \end{array}\right],$$

where $f_{u}$ and $f_{v}$ are the focal lengths of a camera, and $O_{u}$ and $O_{v}$ are the central positions on an image in the *u*- and *v*- axes, respectively. Thus, the camera external parameters, T, to be estimated are expressed as a 4 × 3 consisting of R and t:

(3) $$T=\left[R\;t\right].$$

If we can determine K and T, we can project a 3-D LIDAR point cloud on a 2-D camera image using Equation (1) and perform a data fusion. We estimate the camera intrinsic parameter, K, using well-known checkerboard calibration, and the method can be utilized in some open software [58]. Our proposed extrinsic calibration is employed to estimate only the camera external parameters T. If we provide the known camera intrinsic parameter, K, the camera external parameters are simply derived by solving the PnP problem [59] using the coordinates of p and P in Equation (1).

### 3.2. Calibration Targets

**Figure 1:**
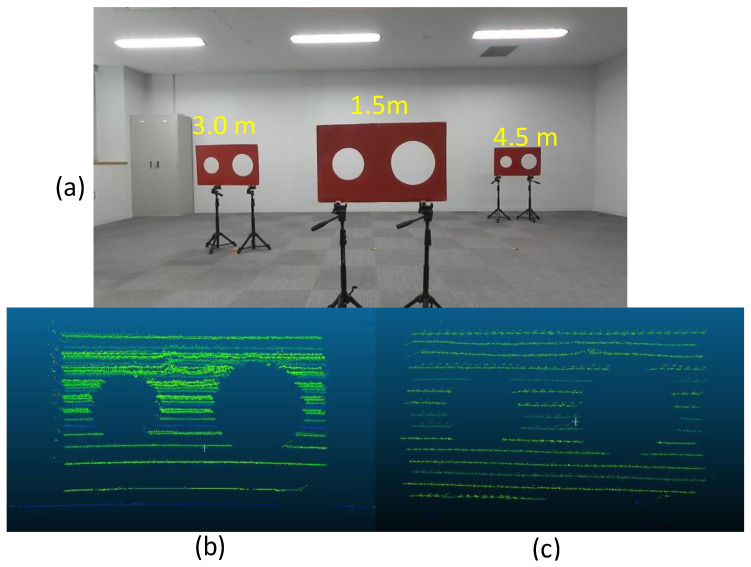
The calibration boards set at various distances in both the (**a**) camera image and the 3-D point cloud taken at distances of (**b**) 1.5 m and (**c**) 4.5 m.

Figure 1 shows the adopted calibration target, which is a red board with circular holes, similar to that employed by Velas et al. [37]. However, the board has only two holes: one small hole (radius: 9 cm) and one large hole (radius: 12 cm), as employed in Yamada and Yaguchi [39]. We observe the target board using the 3-D LIDAR and the RGB camera simultaneously. In this study, we deploy multiple calibration boards while controlling their positions. Figure 1 shows an example where three boards are set at various distances of near (1.5 m), middle (3.0 m), and far (4.5 m). The two circles on each board in both the camera image and the 3-D point clouds are extracted as the feature points for the extrinsic calibration.

### 3.3. Circle Detection

The circles on the board in the camera image shown in Figure 1a can be detected by the Hough transform [60], and the center point and circle radius of each circle are obtained as 2-D pixel values. We calculate the subpixel coordinates of the circumferences at arbitrary steps of the center angle using the center point and radius. Thereafter, we apply the coordinates of the center point and circumferences as the feature points in the calibration.

The 3-D LIDAR point cloud data can be analyzed using the Point Cloud Library (PCL), which is an open-source library. The circle detection in the 3-D point cloud, as shown in Figure 1b,c, is performed based on random sample consensus (RANSAC) functions [61] and [Kd-tree] [62], which is a space-partitioning data structure that stores k-dimensional points in a tree structure that enable efficient range searches and nearest neighbor searches, available in the PCL. These can be used to find correspondences between groups of points and extract a circle shape in the 3-D space. The 3-D coordinates of the center point and radius of each circle are also obtained from the circle detection. Similar to the detected circle on the image, we derive 3-D coordinates of the circumferences of the large and small circles. Here, we also consider the resolution of the 3-D point clouds. As shown in Figure 1b,c, the point clouds obtained at relatively far distances are conventionally sparse, and the circular shape is not perfectly restored. Further, the noise included in the point cloud could interfere with the shape restoration. Although the deployment of multiple calibration targets is important in extrinsic calibration, as described in previous studies [9], the low resolution and point cloud distortion can prevent the usage of the board set at far ranges. Thus, we sample the radii of the small and large circles in the 3-D point cloud by random walk [54], as described in the following section. The optimized circle radii obtained through a global parameter search can generate the appropriate circles in the 3-D point clouds, even if it is highly sparse and error-prone, improving data fusion accuracy.

**Figure 2:**
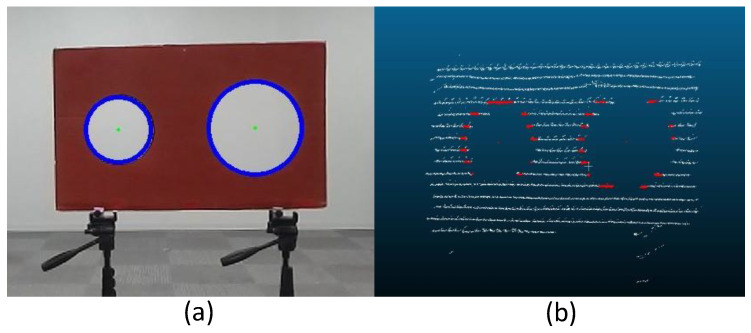
Example of the detected circles on (**a**) camera image (blue line) and (**b**) 3-D point clouds (red line).

In this study, we calculate the coordinates of the circumferences of the circles at a step of 10° of the center angle in both the image and the 3-D point cloud. Thereafter, we obtained 74 data points, consisting of the coordinates of the circumferences and the center points of the small and large circles as the feature points per board. These data points corresponded to the coordinates of p in the image and those of P in the 3-D point cloud in Equation (1). We applied them as input data to estimate the camera external parameters. Although previous studies [10,37] employed calibration boards with four circular holes, we obtained sufficient input data using circle circumference and multiple boards with two circular holes. Figure 2 shows an example of the detected circles on both the camera image and the 3-D point clouds.

### 3.4. Design of the Likelihood Function and Parameter Sampling

We set the 2-D pixel coordinates of the centers and circumferences of the circles extracted from an image as $\left(u_{i}^{c},\;v_{i}^{c}\right)\;\left(i=1,\;N\right).$ N is 222 for the three boards. The 3-D coordinates of those in the point clouds are also defined as $\left(x_{i}^{c},y_{i}^{c},z_{i}^{c}\right)\;\left(i=1,\;N\right).$ When we substitute values for K and T in Equation (1), the 3-D points in the point cloud can be projected onto the image, and we set the 2-D projected points as $\left(u_{i}^{p},v_{i}^{p}\right)$, derived from $\left(x_{i}^{c},y_{i}^{c},\;z_{i}^{c}\right)$. As described in the above section, we vary the radii of the circles by random walk without searching for the camera external parameters directly. This is because the circle detected in the 3-D point clouds may not be perfectly restored because of noise and sparseness. Here, we represent the circle radius as cr. If we apply three boards, as shown in Figure 1a, cr will have six components, corresponding to the radii of the small and large circles of each board for one sampling:

(4) $$cr=[{cr}_{1s}\;{cr}_{1l}\;{cr}_{2s}\;{cr}_{2l}\;{cr}_{3s}\;{cr}_{3l}],$$

where ${cr}_{1s}$ and ${cr}_{1l}$ are the radii of the small and large circles of board 1; ${cr}_{2s}$, ${cr}_{2l}$, ${cr}_{3s}$, and ${cr}_{3l}$ are the radii for boards 2 and 3, respectively, the values of which vary for each sample. Thus, the coordinates of the circumferences of the detected circles in the 3-D point clouds also vary with cr and we represent them as $\left(x_{i}^{cm}\left(cr\right),\;y_{i}^{cm}\left(cr\right),\;z_{i}^{cm}\left(cr\right)\right)\left(i=1,\;N\right)$, including the coordinates of the fixed circle centers. Subsequently, the 2-D pixel values of the projected 3-D circle points are changed as follows: $\left(u_{i}^{pm}\left(cr\right),\;v_{i}^{pm}(cr)\right)\left(i=1,\;N\right)$.

Here, we define the likelihood function, L(cr), based on Masegaard and Tarantola [54] as follows:

(5) $$L\left(cr\right)=\;k\;exp\left(-\alpha \sum_{i}\frac{{\left|P_{i}^{I}-P_{i}^{L}(cr)\right|}^{2}}{2\sigma_{p}^{2}}\right),$$

(6) $$P_{i}^{I}=\left(u_{i}^{c},\;v_{i}^{c}\right)\;\left(i=1,\;N\right),$$

(7) $$P_{i}^{L}(cr)=\left(u_{i}^{pm}\left(cr\right),\;v_{i}^{pm}(cr)\right)\;\left(i=1,\;N\right),$$

where k is an appropriate normalization constant, α is the scaling factor, and $\sigma_{p}$ is the uncertainty in pixel scale. Although the detected circle on the 2-D image is generally accurate in the subpixel scale, we define $\sigma_{p}$ as 1.0 conservatively.

Next, we sample cr and derive a posteriori probability density of the camera external parameters by following the random walk rule:

1. First, we employ the values of the radius extracted from the 3-D circles using PCL, as described in Section 3.3, as the first sample. We define the radius as ${cr}_{currrent}$.
2. We estimate the camera external parameters by solving the PnP problem [59] using ${cr}_{current}$, after which we derive $P_{i}^{L}({cr}_{current})$ in Equation (7). Thereafter, $L({cr}_{current})$ is calculated using Equation (5).
3. We sample the next parameter, ${cr}_{new}$, randomly based on a priori information and derive the camera external parameters using ${cr}_{new}\;$ and $P_{i}^{L}({cr}_{new})$, following which we calculate $L({cr}_{new})$.
4. The acceptance or rejection of ${cr}_{new}$ is assessed using the following probability relation [54,55]:
  (8) $$Pr=min\left[1,\;\frac{L({cr}_{new})}{L({cr}_{current}))}\right].$$
  If the likelihood function, $L\left({cr}_{new}\right)\geq \;L\left({cr}_{current}\right)$, ${cr}_{new}\;$ is accepted, and if $L\left({cr}_{new}\right)<L\left({cr}_{current}\right)$, ${cr}_{new}$ is accepted with a probability of $\mathrm{Pr}\;(L\left({cr}_{new}\right)/L\left({cr}_{current}\right))$.
5. If ${cr}_{new}\;$ is accepted, the camera external parameters derived using the ${cr}_{new}$ are applied to obtain a posteriori probability density, and ${cr}_{new}$ is replaced with ${cr}_{current}$. If ${cr}_{new}$ is rejected, new radii are sampled and ${cr}_{current}$ remains unchanged.
6. We repeat processes 2–5 at a designed count and obtain a posterior probability density consisting of the accepted parameters.

**Figure 3:**
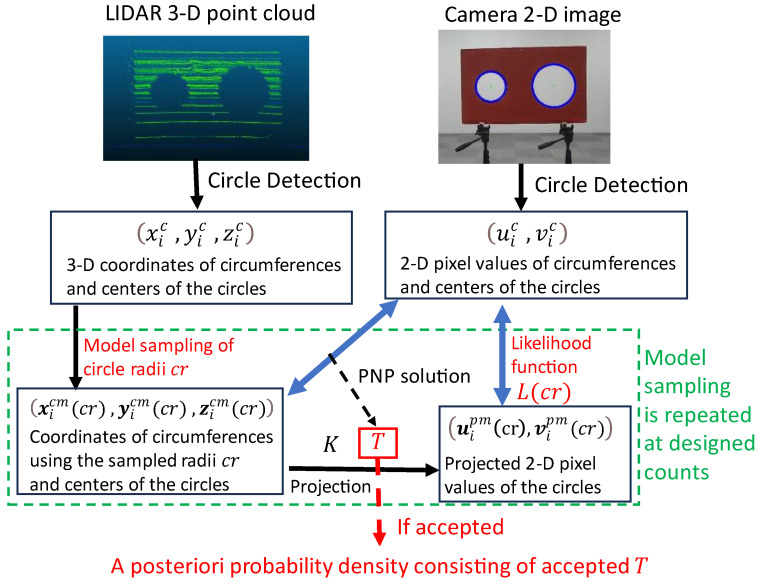
The flow of the derivation of the a posteriori probability density of the camera external parameters.

The flow of the derivation of the a posteriori probability density is summarized in Figure 3. Following this process, the radius of the 3-D circler can be sampled to ensure better data matching between the LIDAR and camera data. The obtained probability density is utilized to select the appropriate camera external parameters.

## 4. Experiments

We describe our measurement system for obtaining data on the camera and the 3-D LIDAR for the extrinsic calibration and the 3-D mapping. Thereafter, we explain the deployments of the calibration targets for investigating the effects of target positions on the data fusion accuracy.

### 4.1. Measurement System

**Figure 4:**
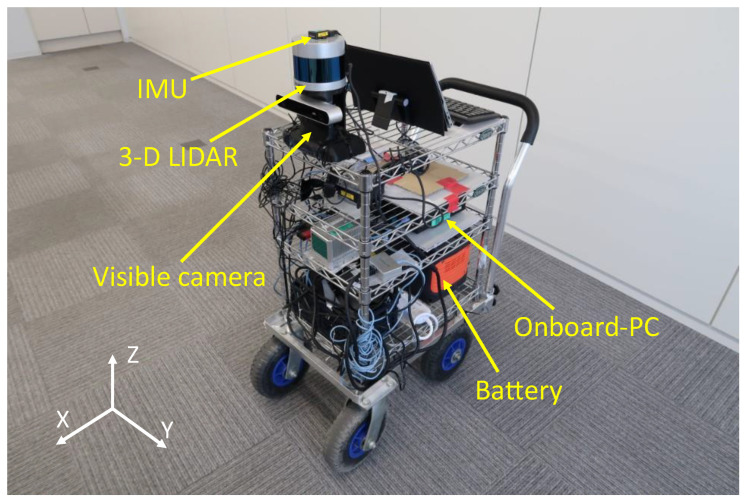
Measurement system used for the extrinsic calibration and 3-D mapping. The coordinate system drawn using white arrows indicates the world coordinate system, corresponding to that of the 3-D LIDAR.

Figure 4 displays the measurement system constructed as a mobile wagon. It has onboard sensors: 3-D LIDAR (VLP-32C (Velodyne LIDAR, SanJose, CA, USA), a visible camera (ZED stereo camera (SteroLabs, SanFrancisco, CA/USA)), and IMU (3DM-GX5-25 (Microstrain by HBK, Williston, ND/USA)). The 3-D LIDAR and the visible camera are rigidly fixed on the wagon, and their measurement data are employed for extrinsic calibration. The 3-D point cloud data and IMU data are employed for the 3-D mapping using the LIDAR-SLAM algorithm described in Section 7. All sensors are controlled by a robot operation system (ROS) installed on the onboard PC (Figure 4), and their data are recorded in ROS format. The sensors and the PC are powered by an onboard battery (Figure 4), and the system on the mobile wagon can be operated in stand-alone mode.

### 4.2. Experimental Setups

**Figure 5:**
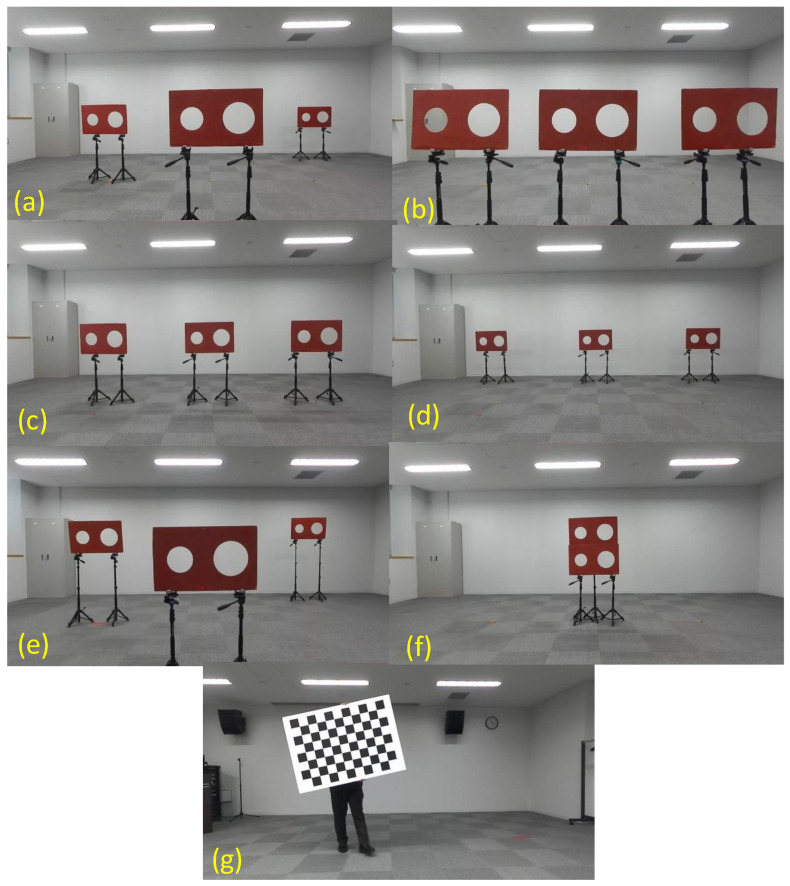
Deployments of the calibration boards applied in this study. (**a**) The three red boards with two circular holes (calibration targets) are deployed at distances of 3.0, 1.5, and 4.5 m from left to right. The calibration targets are deployed at even distances of (**b**) 1.5, (**c**) 3.0, and (**d**) 4.5 m. (**e**) The three boards are set at the same distances as in (**a**) and have different heights of 1.0, 0.7, and 1.3 m. (**f**) Deployment of two calibration targets, one above the other, at a distance of 3.0 m. (**g**) One shot of the extrinsic calibration using a checkerboard.

Table 1 summarizes all the experimental patterns of the extrinsic calibration performed in this study, and Figure 5 indicates the deployments of the calibration boards used. We vary the deployment distances of the calibration boards to investigate the effect of the distance between the sensors and the calibration targets on the estimation of the camera external parameters and the accuracy of data fusion (Figure 5a–d) at the same height (0.7 m). Three boards are deployed at distances of 3.0, 1.5, and 4.5 m from left to right, as shown in Figure 1a and Figure 5a (Case A in Table 1). In Figure 5b–d, three boards are deployed at even distances of 1.5, 3.0, and 4.5 m (Cases B, C, and D). In Case E, the three calibration boards are set at different heights of 1.0, 0.7, and 1.3 m and distances of 3.0, 1.5, and 4.5 m (Figure 5e) to investigate the effect of the height of the calibration target. Figure 5f displays the deployment of two boards, one above the other, at a distance of 3.0 m from the sensors (Case F). This deployment of four circles at a fixed distance is comparable to the setup in previous studies [10,37,38]. As shown in Figure 5g, we also attempted the extrinsic calibration using a checkerboard. In this case, we employed a state-of-the-art method [23] available in the Matlab R2021a open-source software [25] for the extrinsic calibration. For a fair comparison, we use the data on three poses of a single checkerboard taken at approximately 1.5, 3.0, and 4.5 m (Case G), and the derived camera external parameters are referenced for our evaluation. Additionally, to verify the effect of the algorithm, we derive the camera external parameters without using the Monte-Carlo Markov-chain algorithm described in Section 3.4 but using the deployment in Figure 5a (Case H).

## 5. Camera External Parameter Results

For Cases A–F in Table 1, we perform parameter sampling of the radii of the 3-D point cloud circles using random walk and obtain the a posteriori probability density of each component of the camera external parameters. The VLP-32C LIDAR (Velodyne LIDAR, SanJose, CA, USA) used in our experiments has an accuracy of ±3 cm for laser ranging, and we provide a range of 2 σ (6 cm) for the radius sampling as a priori information. Further, any value within the range is sampled with the same probability. Here, we derive the probability densities of the rotational vector components, $R_{x}$, $R_{y}$, and $R_{z}$. These vectors correspond to the rotational angles along the x, y, and z-axes and are directly converted into a rotational matrix in T. Next, the probability densities of the translational vector components, $t_{x}$, $t_{y}$, and $t_{z}$, are also derived. During the parameter sampling, components of the rotational and translational vectors are restricted within a realistic range, i.e., ±2π rad and 1.0 m.

**Figure 6:**
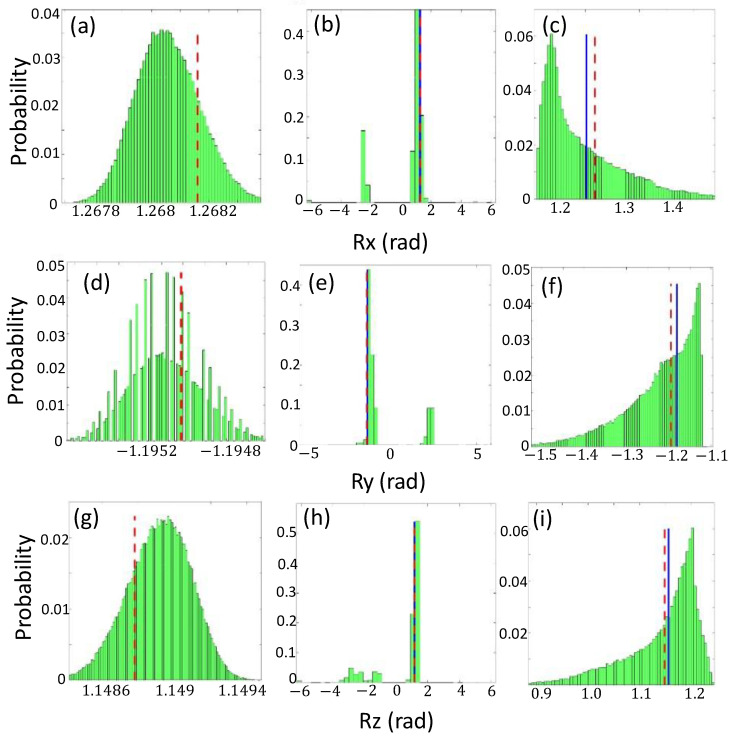
A posteriori probability densities of the rotational vector components: (**a**–**c**) $R_{x}$ for Cases A, B, and F; (**d**–**f**) $R_{y}$ for Cases A, B, and F; and (**g**–**i**) $R_{z}$ for Cases A, B, and F, respectively. The blue and red bold dashed lines indicate the value of each component for Cases G and H.

**Figure 7:**
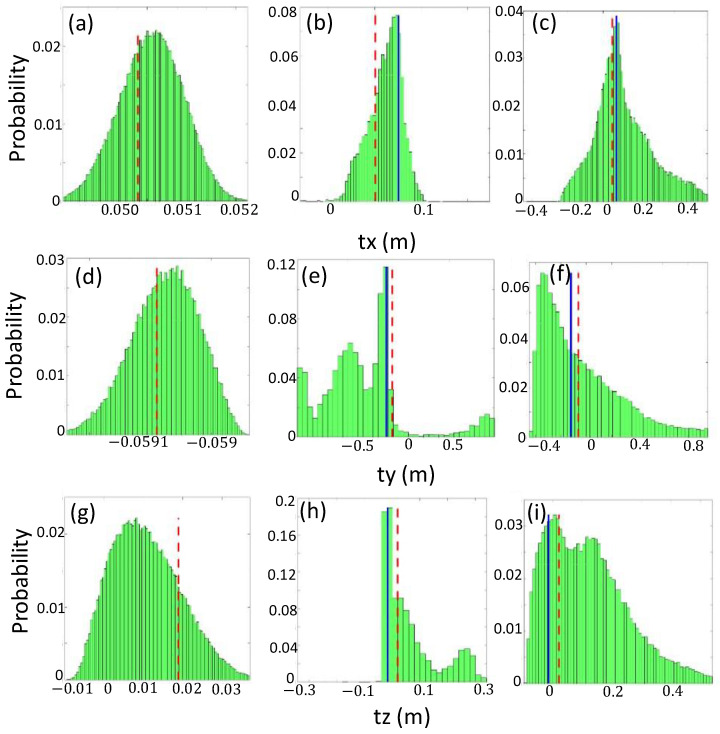
A posteriori probability densities of the translational vector components: (**a**–**c**) $t_{x}$ for Cases A, B, and F; (**d**–**f**) $t_{y}$ for Cases A, B, and F; and (**g**–**i**) $t_{z}$ for Cases A, B, and F, respectively. The blue and red bold dashed lines indicate the value of each component for Cases G and H.

Figure 6 and Figure 7 show the a posteriori probability densities of the rotational and translational vector components for Cases A, B, and F, respectively. In each figure, the derived components of both vectors for Cases G and H are plotted for comparison (the blue bold and red bold dashed lines, respectively). The probability densities encompass the accepted samples during the parameter sampling in 500,000 iterations. In the parameter sampling, we set k=1.0 and vary the scaling factor (α) from $1\times 10^{-5}$ to $5\times 10^{-4}$ in Equation (5), depending on the case. The numbers of the accepted samples are 106,314, 9540, 63, 54, 43,896, and 75,075 for Cases A, B, C, D, E, and F, respectively. The large number of acceptances indicates that the experimental data taken can provide strict constraints on the parameter estimation.

Figure 6 and Figure 7 show the distribution of the ±3 σ range in the most possible bin. Figure 6a,d,g indicate the probability densities of the three components $\left(R_{x},\;R_{y},R_{z}\right)$ of the rotational vector for Case A, and Figure 6b,e,h, as well as Figure 6c,f,i, show those for Cases B and F. The probability densities of the rotation vector for Case A represent almost Gaussian shapes, and the models are sampled within a very sharp range (approximately $9\times 10^{-4}$ rad in 3 σ range of $R_{x}$). Conversely, the models for Case B are sampled as split shapes, although the values in the most possible bin are similar to the results obtained from the nonsampling case (Case H) and the checkerboard calibration (Case G). The deployment of multiple boards at even distances, as in Case B, interferes with the determination of the rotational components, as shown in Figure 6b,e,f. This indicates that the deployment of the target boards set in multiple ranges is more effective in constraining the rotational matrix compared to the deployment at an even range. The probability densities for Case F have shapes that slightly deviate from Gaussian, and the 3 σ range is ~0.5 rad broader than Case A. The utilization of multiple board sets at various distances in contrast to a single board can also effectively constrain the model parameters.

Figure 7a,d,g indicate the probability densities of the three components $\left(t_{x},\;t_{y},t_{z}\right)$ of the translational vector for Case A, whereas Figure 7b,e,h, as well as Figure 7c,f,i, show those for Cases B and F. The probability distributions obtained for Case A also have Gaussian shapes similar to those for the rotational vector, and the deployment in Case A can provide better constraints to sample the translational vector. The translational vector for Case B has been sampled in a narrower range than Case F, differing from the rotational vector. The utilization of multiple boards is effective in constraining the translational vector, regardless of the positions of the calibration targets. Although we do not show the probability densities for Cases C and D due to the small number of acceptances, their distributions are similar to those for Case B, where the multiple calibration targets are set at even distances.

The shapes of the probability density distributions for Case E are similar to a Gaussian, although they are slightly distorted and have a broader 3 σ range compared with those for Case A. For example, the 3 σ ranges of $R_{x}$ are $5.8\times 10^{-3}$ rad for Case E and $9\times 10^{-4}$ rad for Case A. In Case E, the heights of the boards are different from those in Case A, and the 3-D point clouds of the highest board (right board in Figure 5e) are very sparse. This is because scanning lasers of the employed LIDAR are sparse in the upper and lower parts of the sensor. The broader range of the accepted parameters in Case E could be due to the sparseness, and the dense point cloud of a calibration target should provide a strict constraint on the parameter estimation.

In their recent research using a planer board with circular holes, Beltrán et al. [10] highlighted the effectiveness of using multiple poses of the board for extrinsic calibration. In their evaluation, they indicated the differences in the estimated external parameters obtained using their method and the ground truth in a virtual environment (i.e., $8.2\times 10^{-3}$ m in the translation vector and $2.4\times 10^{-4}$ rad in the rotation matrix when three poses were applied). We acknowledge that we cannot compare their results with ours directly because we performed our experiments in real environments, and we do not know the ground truth. However, if we assume the dispersion of the probability density as deviation from the true value, the average 1 σ errors of the translational and rotational components are $5.6\times 10^{-3}$ m and $2.97\times 10^{-4}$ rad for Case A, respectively. The performance of our applied method, using three boards set at various distances, is sufficiently comparable to those of the state-of-the-art methods.

## 6. Evaluation of Data Fusion for the Objects Set at Fixed Positions

We have estimated the camera external parameters for several deployments of the calibration targets. The results showed that the usage of multiple targets set at various distances can provide a strict constraint on the parameter estimation using probability density. Conversely, the effects of the positions of the calibration targets on the data fusion accuracy have not been clearly investigated. In particular, when an object is observed by both a camera and a 3-D LIDAR at a mutually fixed position, it is important to know the appropriate deployment approaches for extrinsic calibration to obtain good data fusion results. In this section, we evaluate the accuracies of data fusion of objects set at fixed positions between the objects and the sensors for each deployment of the calibration targets, as shown in Figure 5. Thereafter, the relationship between the positions of the objects used in the data fusion and the deployment of calibration targets is investigated quantitatively.

In the evaluation, we use a red board as the fixed object and obtain the camera images and 3-D point cloud data using the mobile wagon (Figure 4). The 3-D point cloud can be projected on the 2-D image using the camera intrinsic and external parameters, and each projected 3-D point can obtain the RGB values of a corresponding pixel of the image. Thereafter, a 3-D colored point cloud is generated, and we use the colored data to evaluate the accuracy of the data fusion. If the camera intrinsic and external parameters used in the data fusion are accurate, the colors in the 3-D colored point cloud can be restored perfectly in noise-free environments. Therefore, we measure the red color ratios of the red boards in the 3-D colored point cloud as the data fusion accuracy for each calibration listed in Table 1.

As shown in Figure 6 and Figure 7, the camera external parameters are obtained as probability densities except for Cases G and H. For the evaluation, we extract the parameters corresponding to the 10 largest likelihoods, L(cr), in Equation (5) from the probability density distribution and calculate the red color ratios using the 10 selected camera external parameters for Cases A–F.

### 6.1. Experimental Setup

**Figure 8:**
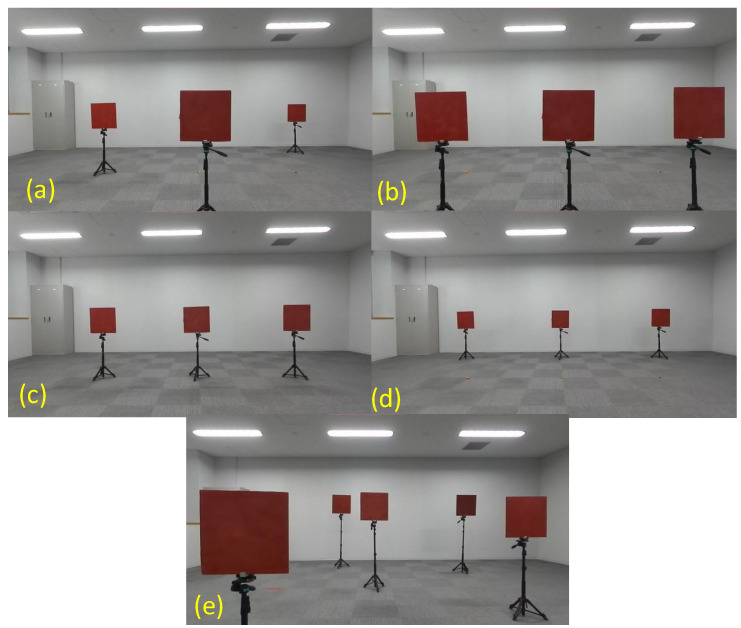
Deployment of the 2-D red boards used for the evaluation of data fusion. Three red boards are deployed at distances of (**a**) 3.0, 1.5, and 4.5 m; (**b**) 1.5 m and (**c**) 3.0 m; and (**d**) 4.5 m, respectively. (**e**) The five red boards are deployed at various positions: distances of 1.0, 4.5, 3.0, 4.0, and 2.0 m and heights of 0.7, 1.2, 1.0, 1.1, and 0.8 m.

We deploy the three red square boards with each size of 0.4 m, corresponding to the deployment of the calibration targets in Cases A, B, C, and D, as shown in Figure 5. This is because we can investigate the relationship between the positions of the objects used in the data fusion and those of the calibration targets. As shown in Figure 8, three red boards are set at distances of 3.0, 1.5, and 4.5 m from left to right (Figure 8a) and 1.5 m (Figure 8b), 3.0 m (Figure 8c), and 4.5 m (Figure 8d). These deployments of the red square boards (Figure 8a–d) correspond to those of the calibration targets shown in Figure 5a–d, respectively. Then, we have also deployed the five red boards at various distances and heights. They are spread out in the front field of view of the camera and the 3-D LIDAR (Figure 8e). It is also useful to evaluate the practical adaptability of each calibration pattern if the objects are deployed at various positions.

### 6.2. Evaluation Results

**Figure 9:**
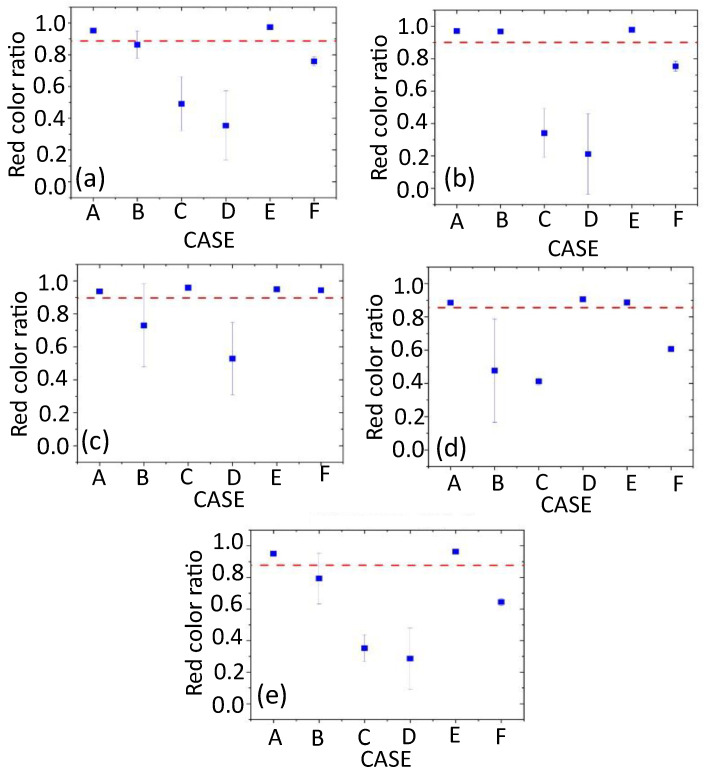
The average red color ratios of the red boards in the 3-D colored point cloud for Cases A–G. Each figure (**a**–**e**) represents the color ratios corresponding to the deployment of the red boards in Figure 8a–e, respectively. The blue square point in each figure indicates the average and standard deviations of the average red color ratios of the boards for 10 selected camera external parameters in each case. The red dashed horizontal line indicates the red color ratio obtained in Case G.

Figure 9a–e show the averages of the red color ratios of the three or five red boards, corresponding to the deployment shown in Figure 8a–e for Cases A–G, respectively. As described above, the red color ratio has been calculated using the 10 selected camera external parameters, and the average and standard deviations of the average red color ratio are represented as a blue square point in Figure 9. The red dashed horizontal line in each figure of Figure 9 indicates the red color ratio obtained for Case G (checkerboard calibration).

Figure 9 indicates that a high red color ratio is achieved when the positions of the red boards and the calibration targets are consistent. For example, when the red boards are set at a distance of 1.5 m, a high red color ratio is achieved in Case B (Figure 9b), where all calibration targets are located at 1.5 m (Figure 5b). Similarly, Figure 9d shows that the red color ratio is high when the red boards are located at 4.5 m and the calibration boards are set at 4.5 m (Case D: Figure 5d). This indicates that the calibration target set at a suitable distance from the sensors can yield appropriate camera external parameters, enabling the effective data fusion of objects set at similar positions. Contrarily, extrinsic calibration using targets set at a fixed distance (Cases B, C, D, and F) can be less effective for the data fusion of an object deployed at other distances, as shown in Figure 9. In that case, the red color ratio also has a high standard deviation. The calibration using the targets set at multiple distances (Cases A and E) is useful for achieving high red color ratios and low standard deviations for all deployments (Figure 9). The red color ratios obtained from the calibration results in Case E are slightly higher (~1–2%) than those in Case A for all deployments, as shown in Figure 9. Since the calibration boards in Case E are deployed with varying the heights, the height variation may be slightly useful for obtaining appropriate external parameters in the various deployments for data fusion.

The red color ratios derived from the calibration using the checkerboard (Case G) also indicate high values for all deployments (red dashed line in Figure 9). This is because the checkerboard data are also obtained at three positions (distances of ~1.5, 3.0, and 4.5 m). However, the red color ratios for Cases A and E and those derived from the measurements where the red boards and the calibration targets are located at the same positions are still higher than those for Case G. This indicates that the calibration using circle detection as the calibration target is more effective than the checkerboard calibration and/or effectiveness of the global parameter sampling using the probability-based method.

The red color proportions for Case F (Figure 5f) are approximately 60–75%, except for when the calibration targets are located at a distance of 3.0 m (Figure 9). These results are better than those for Cases B, C, and D, where all calibration targets are set at even distances. As described in Section 5, the calibration using the multiple boards set at even distances can impede the determination of the rotational components, resulting in a large deviation in the probability densities of the rotational vector (Figure 6b,e,h). Thus, the utilization of multiple boards cannot necessarily generate better projections compared to the single board case if we do not consider appropriate deployment.

We do not indicate the red color ratios for Case H in Table 1 because the values are only slightly lower than those for Cases A and E. Conversely, when we applied the fixed radii (12 cm and 9 cm for the large and small circles, respectively) without performing model sampling for other cases, we could not obtain any camera external parameters for decent projections. Thus, we establish that the selection of appropriate radii for generating 3-D point cloud circles using the probability method is important for improving the data fusion accuracy.

**Figure 10:**
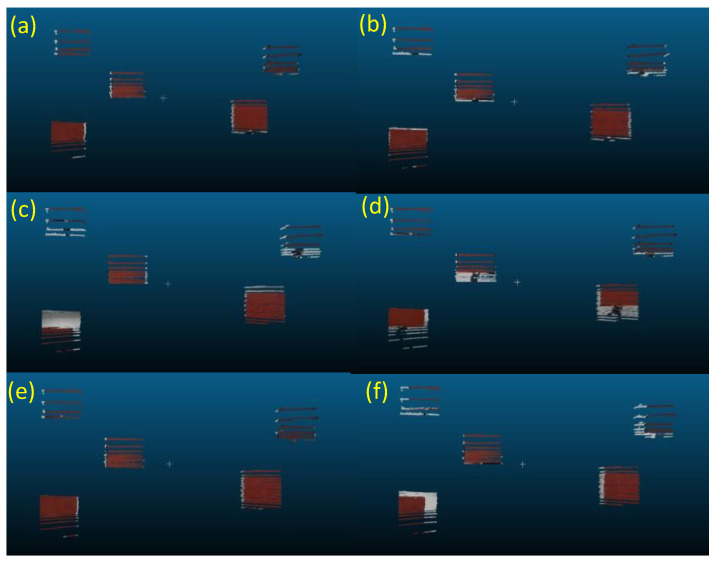
The 3-D colored point cloud of the five red boards, as shown in Figure 8e. (**a**–**f**) The results of the best data fusion obtained for Cases A–F, respectively.

Figure 10 indicates examples of the 3-D colored point cloud for the deployment of the red boards shown in Figure 8e. These figures show the best data fusion results for Cases A–F, respectively. We can also observe a clear relationship between the accuracy of data fusion and the deployment of the calibration targets. Although the extrinsic calibration using boards set at far distances (Case D) (Figure 5d) is useful for performing the data fusion of objects set at similar distances, it is ineffective for objects at near distances (Figure 10d). Similar tendencies are also observed for Cases B, C, and F (Figure 10b,c,f). In more practical situations where multiple objects are deployed at various positions, as shown in Figure 8e, we can determine the effectiveness of deployments of multiple calibration targets set at various distances and heights as Cases A and E. 

## 7. Evaluation of Data Fusion in 3-D Mapping

Finally, we propose an evaluation method using the color ratio in a 3-D mapping scenario. We construct a 3-D data fusion map with a scale of a few tens of meters using the LIDAR-SLAM algorithm in an outdoor environment. This is close to a more realistic scenario, and the evaluations of the obtained camera external parameters are appropriate for assessing their practicality and the applied camera extrinsic calibration methods.

### 7.1. Experimental Setup

**Figure 11:**
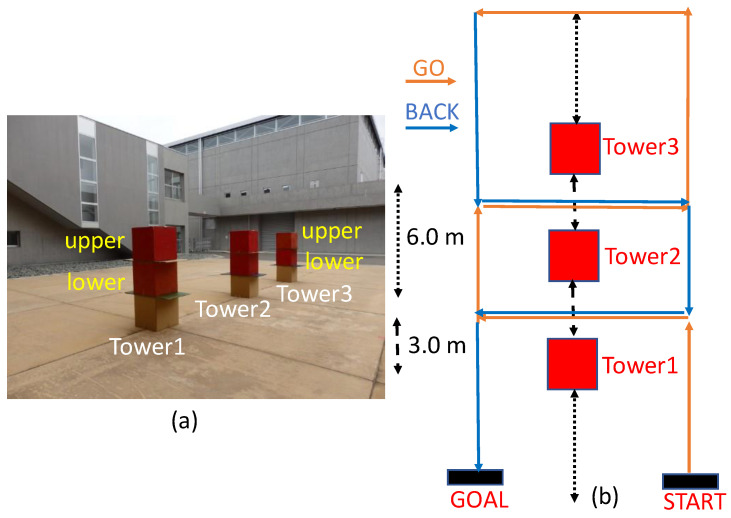
(**a**) Photograph of the deployed three box towers in the court of the Fukushima Robot Test Field. (**b**) The top view of the route of the movable wagon around the three towers. The orange and blue arrows indicate the travel direction of the wagon.

We prepared nine red cube boxes with side lengths of 50 cm and built three box towers, each consisting of three cube boxes. In each box tower, the top two boxes are painted red. We deployed the three towers in the court of the Fukushima Robot Test Field, as shown in Figure 11a. The top two red boxes in each tower are denoted as “upper” and “lower” chronologically (Figure 11a). Next, we obtained both the camera image and 3-D point clouds of the three box towers using the running mobile wagon (Figure 4). As shown in Figure 11b, the wagon moved around each tower, and we could obtain data on all four sides of the six red boxes.

We constructed a 3-D point cloud map including the three towers using the LIDAR-SLAM algorithm (LIO-SAM [3]). Since we obtained timestamps of each 3-D point cloud data and camera image simultaneously, we constructed a 3-D colored point cloud map by projecting a 3-D point cloud on the synchronized 2-D color image using the timestamp data. For the data fusion, we applied the camera external parameters obtained for Cases A–G and determined the appropriate case for the 3-D data fusion using the red color ratio of the six red cube boxes. Although we evaluated the red color ratios for the targets located at the fixed positions in the previous section, we employed the data obtained at various distances and heights in this 3-D evaluation as the realistic situation.

### 7.2. Evaluation Results

**Figure 12:**
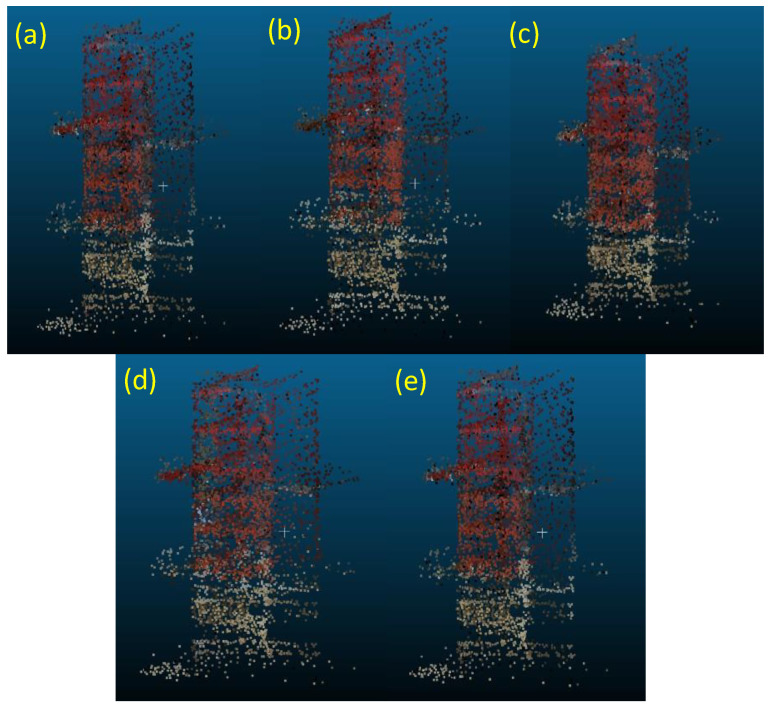
The 3-D colored point cloud of Tower 1 (Figure 11). (**a**–**e**) The result of data fusion using a selected camera external parameter derived from Cases A, B, E, F, and G, respectively.

**Figure 13:**
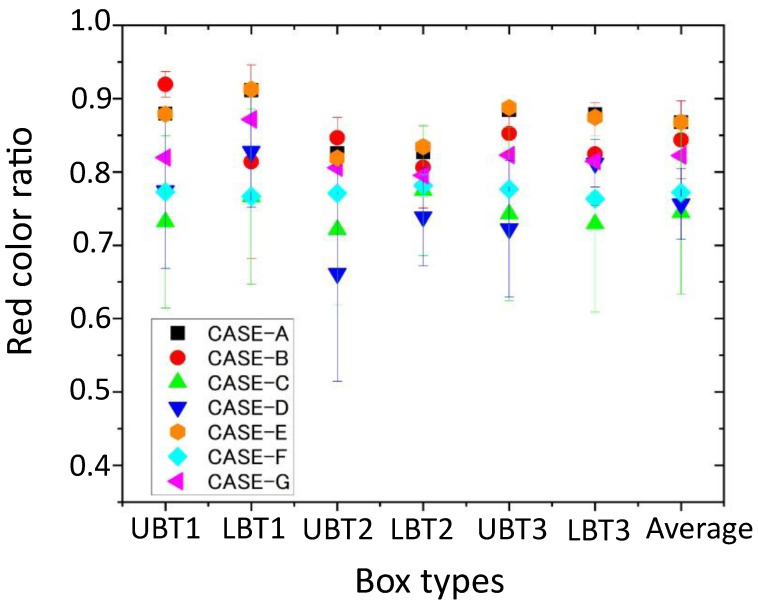
The red color ratio of each box type and the average for Cases A–G. “UBT1” and “LBT1” denote the upper and lower box of Tower 1, respectively. “UBT2”, “LBT2”, “UBT3”, and “LBT3” denote the upper and lower boxes of Tower 2 and 3, respectively. “Average” indicates the average of the red color ratios of the six boxes for each case.

We constructed a 3-D colored point cloud map consisting of approximately 3700 point cloud frames, including the three box towers. In this evaluation, we applied the same camera external parameters employed in the evaluation in Section 6, especially the 10 selected parameters for Cases A–F. Figure 12 shows examples of the 3-D colored point clouds of Tower 1 in the constructed map for Cases A, B, E, F, and G. Figure 13 shows the red color ratios of the six red boxes constituting the three towers, i.e., the upper and lower red boxes of Towers 1, 2, and 3, as well as the average of the six boxes for Cases A–G. The red color ratios in Figure 13 for Cases A–F indicate the average and standard deviations of the values derived using the 10 selected camera external parameters.

Figure 12 and Figure 13 indicate that the highest red color ratios are achieved for Cases A and E, similar to the results shown in Figure 9. In Figure 12, although the red colors of the upper and lower boxes for all cases are almost completely restored, some misprojections and false colors are included. Such wrong projections are not significant for Cases A and E. The red color ratios for Cases C, D, and F have values of ~70–80%, and these calibrations (Figure 5c,d,f) are less effective for constructing 3-D data fusion map compared with those in other cases. The red color proportions for Cases B and G are also over 80%. In the calibration of Case B, all calibration boards are set at a near distance of 1.5 m, and dense point clouds are obtained, in contrast with those obtained for further targets. This indicates that the density of the point cloud of the calibration target is also important for obtaining good external parameters, as described in Section 5.

Notably, it is difficult to identify the clear relationship between the distances at which the data are taken and those where the calibration targets are located because the 3-D colored map includes the data taken at various distances and heights. Additionally, we cannot find a significant difference between the average red color ratio for Case A and Case E. The variations in the heights of the calibration targets may not considerably affect the data fusion accuracy in the complex combined 3-D case due to the sparseness of the point cloud of the high calibration targets. However, the variation in the distances of multiple calibration targets is vital for effective data fusion in realistic situations. 

## 8. Discussion

In this paper, we first clarified the relationship between the positions of the calibration targets and those of objects in the data fusion map quantitatively. The extrinsic calibration implemented using targets set at fixed distances from the sensors is useful for obtaining the camera external parameters for achieving good data matching for the objects located at similar distances. However, the utilization of calibration targets at fixed distances is ineffective for obtaining the parameters that provide strict constraints on the data fusion of the objects located at other distances. The results of the extrinsic calibrations in Cases B, C, and D (Figure 6 and Figure 7) indicate that the utilization of multiple calibration targets set at even distances is effective for estimating the translational vector components. However, this approach impedes the estimation of the rotational vector components. Therefore, we establish that extrinsic calibration using multiple calibration targets set at various distances is a superior method.

Notably, the best data fusion accuracies are obtained in Cases A and E, where multiple targets are deployed at various distances. These results are superior to those obtained using the recent checkerboard calibration method (Case G) [23] and comparable to those obtained using a calibration board with circular holes [10]. In a previous study where circular shapes [10] were explored, only a single board with four circular holes was employed. While the researchers successfully improved the estimation accuracy of the external parameters using multiple poses, additional information, such as AR marker and multiple settings, was required. Our method uses only single-view data of the multiple simple boards, and no additional analysis and targets are required. A comparative study between our method (Case A) and the checkerboard calibration method (Case G) highlighted the usefulness of the board with circular holes as the calibration target and/or effectiveness of the Monte-Carlo Markov-chain method. The automatic checkerboard detection algorithm in the Matlab R2021a open-source software [25] often omits information. Then, occasionally, the edge of the checkerboard is not perfectly captured because of the sparseness of the 3-D point cloud [32]. Consequently, the algorithm may generate some errors in the derivation of the camera external parameters. Circle detection could be a more robust method of extracting feature points for the calibration. In this study, we deployed only three calibration targets at distances of 1.5, 3.0, and 4.5 m (Case A) and several heights (Case E). The automatic detection of targets with circular shapes whose positions are numerously changed towards the sensors should be a refined method for achieving high-accuracy data fusion.

Through the data analysis of the extrinsic calibration data using the Monte-Carlo Markov-chain algorithm, the probability distribution of the camera external parameters can be derived. The probability distribution shows the configurations of the extrinsic calibration that are effective for constraining each component of the rotational and translational vectors. Although we establish the effectiveness of multiple targets set at various distances, in practice, a target shape set at a far distance in the LIDAR 3-D point clouds cannot be perfectly restored because of sparseness and noise. Thus, our global sampling of the target shape, such as the circle radius, using the probability-based method can be applied to restore the appropriate shape in the sparse point cloud and obtain the optimal external parameters. While it is difficult to obtain the appropriate parameter when we apply fixed circle radii, as described in Section 5, our proposed probability-based method can largely improve the estimation of the external parameters. The global parameter sampling using the Monte-Carlo Markov-chain algorithm for the optimization of the model parameters should be incorporated into the several extrinsic calibration methods.

Additionally, we proposed an evaluation method based on the color ratio in the 3-D data fusion map. This is similar to a realistic, complex scenario, and the evaluation can be useful for verifying, practically, the adaptability of the camera external parameters in relation to data fusion. In this evaluation, we applied only the red color ratio. If we applied different colors and reflectors for the evaluation, the absolute values of the restoration might have changed. Conversely, the relative accuracy for each calibration method remains constant when a common benchmark, such as red color, is applied. Future studies can explore the appropriate benchmarks for this 3-D evaluation.

## 9. Conclusions

In this study, we investigated concepts related to the deployment of the targets in LIDAR–camera calibration, parameter search in the analysis of the calibration data, and the evaluation method of data fusion accuracy. We established a clear relationship between the position of the calibration targets in the extrinsic calibration and those of the objects used in data fusion. Additionally, we demonstrated the value of the global parameter search using the Monte-Carlo Markov-chain algorithm for the calibration data encompassing sparse and noisy point clouds. Finally, we developed a method for evaluating the obtained camera parameters using a 3-D data fusion map. Based on our research, we propose the following as key points for achieving good extrinsic calibration:

- Usage of multiple targets or poses set at various distances.
- Matching the positions of the calibration targets to the object positions in data fusions, if the latter positions are determined.
- Usage of the dense LIDAR point cloud of the calibration target.
- Restoring the shapes of the calibration target to a circular shape using appropriate parameter control.

These concepts are important for LIDAR–camera calibration. Finally, our deployment approaches for the calibration targets (as in Cases A and E) (Figure 5a,e) and the parameter search using the probability-based method yielded good estimations of the camera external parameters and a high data fusion accuracy. However, perfect data fusion remains elusive, as shown in Figure 13. Future works concerned with the automatic detection of multiple targets, fine adjustment of target shapes, and data acquisition will yield improved data fusion results.

## Figures and Tables

**Table 1:** Experimental patterns of the extrinsic calibration.

	Conditions
Case A	Three boards set at distances of 3.0, 1.5, and 4.5 m (Figure 5a)
Case B	Three boards set at a distance of 1.5 m (Figure 5b)
Case C	Three boards set at a distance of 3.0 m (Figure 5c)
Case D	Three boards set at a distance of 4.5 m (Figure 5d)
Case E	Three boards set at distances of 3.0, 1.5, and 4.5 m and heights of 1.0, 0.7, and 1.3 m (Figure 5e)
Case F	Two boards, one above the other, set at a distance of 3.0 m (Figure 5f)
Case G	Checkerboard taken at three poses at ~1.5, 3.0, and 4.5 m (Figure 5g)
Case H	Same as Case A. The radii of the boards are fixed, and parameter sampling is not performed

## Data Availability

The data presented in this study are available on request from the corresponding author.
